# Inadequate stakeholder management and its effect on a coherent sinkhole risk management strategy: The case of the Merafong Local Municipality, South Africa

**DOI:** 10.4102/jamba.v8i1.265

**Published:** 2016-09-30

**Authors:** Tshepo Moshodi, Christo Coetzee, Kristel Fourie

**Affiliations:** 1 African Centre for Disaster Studies, North-West University, South Africa

## Abstract

The Merafong Local Municipality (MLM) has historically suffered financial and human losses because of the presence of dolomite and the consequent formation of sinkholes. There is a great need for the MLM to address the risk posed by sinkholes to ensure the continued safety of communities. However, as the risk is so pervasive, the MLM needs to coordinate their risk reduction strategies with a wide array of stakeholders in the municipality. Efficient stakeholder management is thus crucial if the sinkhole risk is to be addressed appropriately. This article reviews the current status of stakeholder management in the MLM as it pertains to the formulation of a holistic sinkhole risk reduction strategy. Findings indicate that there are serious deficiencies in the MLM’s stakeholder management relating to key risk management processes such as community involvement in risk management structures, disaster risk assessment, training and awareness, and early warning and response. Improved stakeholder management could be characterised by the following factors: improved two-way communication between the municipality and community stakeholders, fostering a relationship based upon trust and equality amongst stakeholders, participation by a wide array of stakeholder groups affected by the sinkhole risk and a mutual commitment by all stakeholders to address the risk. These factors could contribute to enhancing current and future sinkhole risk reduction strategies.

## Introduction

The Merafong Local Municipality (MLM) has a long history of human and economic losses caused by sinkhole formation because of the presence of dolomite in the region. The presence of this risk puts a sizeable responsibility on the municipality to manage the risk in its entirety. The latter is done by ensuring that institutional structures are put in place, risks are assessed, training and awareness projects are conducted to increase knowledge of the risk amongst key stakeholders, and early warning and response strategies are formulated to mitigate possible impacts. Additionally, there is an increasing urgency placed upon government institutions (at local, provincial and national levels) by international policy documents such as the Hyogo Framework for Action (HFA) (and its successor document, the Sendai Framework for Disaster Reduction) and national legislation such as the *South African Disaster Management Act* to integrate a wide array of stakeholders in activities that aim to address disaster risk. Although the task is daunting, municipalities such as the MLM can make great strides to address all these issues through effective stakeholder management. Stakeholder management is crucial within a disaster management context because it not only allows disaster management entities to gain a better understanding of the risk faced by communities but also facilitates a greater level of trust, communication and participation between stakeholders (UNISDR 2005:25; Van Niekerk & Coetzee 2012:334–336). This in turn greatly improves the possibility of reducing disaster losses.

In light of the crucial role that stakeholder management plays in ensuring multiple stakeholder involvement in disaster risk management, the article will evaluate current efforts of the MLM in managing its stakeholders, with a view to reducing the risk of sinkhole formation within its jurisdiction. The evaluation will focus specifically upon the current state of stakeholder involvement as it pertains to institutional arrangements for risk reduction, disaster risk assessment, training and awareness building, and early warning and response strategies. To gain the necessary insights on all these issues, ward councillors, community members (affected by dolomite and at risk of sinkhole formation) and municipal officials responsible for disaster risk management in the MLM were consulted. Before an evaluation can be conducted, it is necessary to create the risk context which the MLM faces.

## Dolomite and the resulting sinkhole disaster risk within Merafong Local Municipality: An historic overview

**FIGURE 1:**
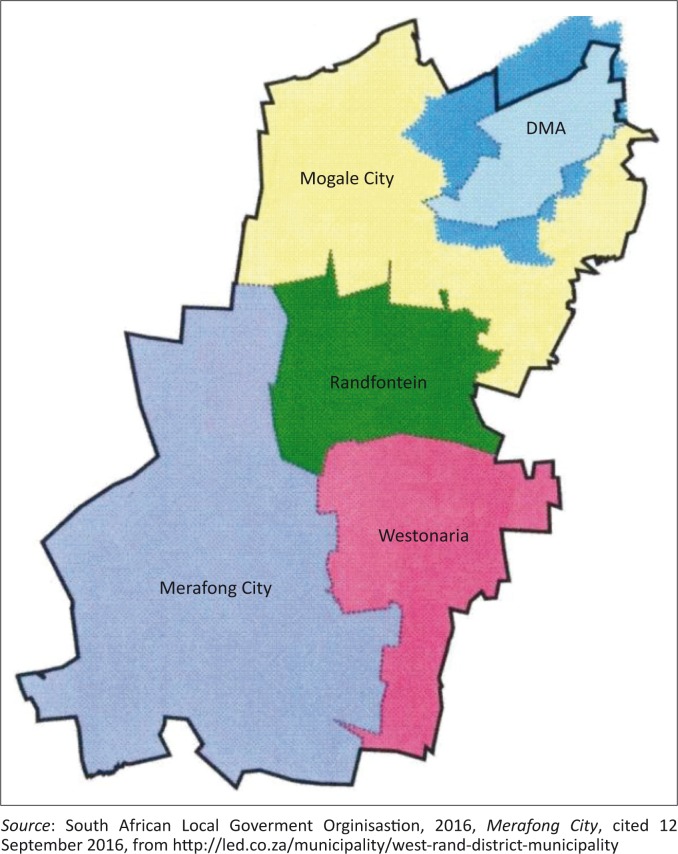
Merafong Local Municipality, Gauteng Province, South Africa.

The MLM is located within the West Rand District Municipality (WRDM), in the Gauteng province of South Africa (see Figure 1). This district is known to be underlain by extensive dolomite rock formations. Dolomite is a soluble carbonated bedrock (Swart *et al*. 2003:753; Zhou & Beck 1997:50). The constant exposure of dolomite to rainwater as well as human-related activities, such as ground water extraction, leakage from water pipes and sewerage systems, dissolves the rock over time because these substances penetrate through the joints of the rock to form openings beneath the surface – which may result in sinkholes (Swart *et al*. 2003:754; Van Eeden *et al*. 2003:97; Zhou & Beck 1997:50). Sinkholes are either caused by the hollowing out or formation of a void below the earth’s surface as a result of normal geological processes, or they may have anthropogenic causes as indicated above. Furthermore, anthropogenic causes such as the construction of roads, township development and associated services, groundwater extraction and groundwater recharge may also give rise to the formation of sinkholes (Buttrick *et al*. 2011:1130; Buttrick &Van Schalkwyk 1998:1; Gutierrez & Guerrero 2008:995; Ngcobo 2006:253; Swart *et al*. 2003:760).

Sinkholes have through the years had a dramatic impact upon the social, economic and infrastructural elements in Merafong. Notable examples include the deterioration of railway facilities in the bank farming area because of unstable ground surface which affected the transportation of goods to and from other towns. This caused consumers to take their businesses away from Merafong to the nearby towns (Ngcobo 2006:253; Swart *et al*. 2003:755; Van Eeden 2003:118). A number of roads have also been affected by the sinkhole risk. Notably, in 1963 both the P89/1 route to Pretoria and the P111/1 route to Johannesburg and some district roads were temporarily closed because of ground instability of the areas and the occurrence of sinkholes (Kirsten *et al*. 2009:26; Ngcobo 2006:255; Swart *et al*. 2003:756; Van Eeden *et al*.2006:410). On another occasion, the business sector of Khutsong township (located near Carletonville) also collapsed into a sinkhole – resulting in financial losses and property damage (Ngcobo 2006:254; Van Eeden 2006:411; Van Eeden *et al*. 2003:99). Van Eeden (2003:116–122) further highlights a number of impacts upon the local business sector associated with sinkholes. These impacts include the disruption in town development, with fewer commercial and residential buildings being erected. In some instances existing building developments had to be destroyed to avoid further risk exposure as was the case in some areas of Carletonville, such as Extensions no 5 and 8. Peri-urban communities in the MLM have also suffered from the presence of sinkholes with some farmers in the rich farming area of bank having to cease with production because of unstable surfaces caused by ground movement. Additional to the financial and infrastructural losses, the impact of sinkholes has also been observed in terms of the loss of life. The highest recorded loss of life in the area because of sinkhole formation occurred when the West Driefontein three-storey crusher plant disappeared into a sinkhole with 29 occupants in 1962 (Swart *et al*. 2003:759; Van Eeden 2003:101). In 1964, two houses and parts of two other houses disappeared into a sinkhole with a loss of five lives at Blyvooruitzicht mining village. To date the available statistics indicate that 35 people have died as a result of sinkholes in the Merafong Municipality (Buttrick & Van Schalkwyk 1998:1–2; Swart *et al*. 2003:102).

What should be clear from this contextualisation is that the MLM has historically suffered very real financial and human losses because of the presence of dolomite and the consequent formation of sinkholes. It is important to note that the problem of dolomite and possible risk of sinkhole formation has not disappeared with time and has in actual fact become worse because of the increased pressures of population growth, land tenure and pressure to provide basic services like water and sanitation (Moshodi 2014:1–109). There is a great need for the MLM to address the risk posed by sinkholes to ensure the continued safety of communities. However, as the risk is so pervasive, the MLM needs to coordinate their risk reduction strategies with a wide array of stakeholders in the municipality. Consequently, efficient stakeholder management would be crucial to address the problem. The article will review the current status of stakeholder management in the MLM as it pertains to the formulation of sinkhole risk reduction strategies, but to do this, stakeholder management theory will first need to be elaborated upon.

### Stakeholder management theory: A broad perspective

The corporate sector has relied greatly on stakeholder management theory since the early 1960s. A key factor that lead to the development of the theory was that business organisations do not exist in a vacuum and that stakeholders that are directly and indirectly affected by the activities of a company can greatly influence the extent to which a company is financially and socially successful. Consequently, stakeholder management theory was developed with the view to explaining how improvements in managing stakeholder relationships could lead to the long-term survival of companies, and possibly higher income for companies, whilst also introducing the notion that stakeholder relationships that do not necessarily only have purely economic benefits to a company, also allow companies to engage in acts of altruism that are crucial for the continued success of a company (Freeman 1984:25–31; Lewis, Hamel & Richardson 2001:6; Payne, Ballantyne & Christopher 2005:85). Within stakeholder management theory the term stakeholder refers to ‘any individual or group who can potentially influence or be influenced by the extent to which an organisation achieves its financial and social goals’ (Daugherty 2001:389–402; Fill 2005:205; Freeman 1984:25; Jahansoozi 2006:942; Lewis *et al*. 2001:6–16). As argued by Freeman (1984:42) and Jahansoozi (2006:943), organisations need to be effective to be successful and to do this they depend upon the resources and support from stakeholder groups. Additionally, the perception that stakeholders have of an institution or organisation greatly influence how they will behave towards it (Hutt 2010:182; Luoma-aho 2006:3). If stakeholders hold a negative perception of an institution, they will behave negatively towards it, the opposite is also true; if stakeholders have a positive perception of an institution their behaviour towards the institution will be positive. A positive perception and behaviour – in effect a good relationship – allows the institution to enjoy the cooperation and support of their stakeholders, which give the institution access to the resources these groups hold for them to achieve its mission successfully (Luoma-aho 2006:3).

In order for an organisation to establish positive relationships with stakeholders, it needs to understand how these stakeholder groups perceive the organisation, how they perceive their environment and understand what each stakeholder group’s needs are (Lewis *et al*. 2001:6). Once the organisation gets to know their stakeholders and pays attention to their needs, a good relationship can flourish with attributes such as mutual satisfaction, commitment from both parties and most importantly, trust (Jahansoozi 2006:943). Therefore, an organisation that considers the relationship it has with its stakeholders as important, and nurtures this relationship, will be successful in achieving its organisational goals (Phillips 2006:35). It is crucial that a relationship is developed where an organisation understands its stakeholders and the stakeholders understand the organisation (Fill 2005:192). In order to create such a relationship of mutual understanding, it is necessary for an organisation to enter into dialogue with its stakeholders (Fill 2005:192). Communication in the form of dialogue, that allows for mutual involvement and input without unequal power relations between stakeholders, can be used as a means to effectively manage and build a relationship. In this way an organisation can also ensure and maintain its credibility and legitimacy (Lewis *et al*. 2001:6). Furthermore, it allows stakeholders to participate more easily in decision-making processes, especially where their interests are concerned (Jahansoozi 2006:943).

The need to manage key stakeholders is not limited to the private sector, in fact, the need to manage stakeholders is even more relevant when one refers to the role of the public sector (Fill 2005:12; Hutt 2010:182). To achieve the developmental goals within a society, governments have to cooperate closely with stakeholder groups such as communities, non-governmental organisations (NGOs), civil society, business and other organs of state. This is especially true for public sector entities dealing with disaster risk management. Because of the complex nature of disaster risk management, it is necessary to have a multisectoral approach to address vulnerability to disaster and consequently to have various individuals involved in this process. (Stanganelli 2008:94; Twigg 2007:6; Vermaak & Van Niekerk 2004:556). The need for multisectoral involvement in disaster risk management is also emphasised by international policies like the HFA (replaced by the Sendai Framework, March 2015) and the *South African Disaster Management Act*. The HFA in particular recognised that any attempts towards integrating disaster risk management into developmental policies, planning and programming for risk reduction, as well as the establishment and strengthening of disaster management institutions, are destined to failure without cooperation between government, civil society organisations, academic institutions and at-risk communities (UN/ISDR 2005:1; Von Oelreich 2011:4; Walker 2007:103). The establishment of mechanisms and capacities to improve resilience to hazards and the incorporation of risk reduction initiatives into preparedness, response and recovery programmes would similarly not succeed without such cooperation. The *South African Disaster Management Act* echoes these sentiments by emphasising that disaster risk management is inherently a:

multi-sectorial, multidisciplinary process of planning and implementation of measures that aim to prevent or reduce the risk of disaster, mitigating the severity or consequences of disasters, emergency preparedness, a rapid and effective response to disasters and post disaster recovery and rehabilitation. (South Africa 2003; Van Niekerk 2005:98)

Both these documents thus acknowledge that a wide array of stakeholders is needed, in order to achieve the critical outcomes of both policies (critical outcomes of both policies are summarised in Table 1).

**TABLE 1:** Policy outcomes Hyogo Framework for Action and *South African Disaster Management Act*.

Policy	Outcome 1	Outcome 2	Outcome 3	Outcome 4	Outcome 5
Hyogo Framework for Action	Making disaster risk reduction a priority	Enhancing knowledge about the risk and taking action	Establish understanding and awareness	Eliminate risk	Be prepared and ready to respond
*South African Disaster Management Act*	Establishing integrated institutional arrangement	Disaster risk assessment	Disaster risk reduction	Response and recovery	-

Stakeholders in terms of disaster risk management refer to those groups who are recipients or targets of policy programmes, risk reduction or development initiatives (Hutt 2010:182; Petkus 2001:27; Rho 2009:8). For risk reduction and management activities these stakeholder groups can include different organs of state (local, provincial, national government or line departments), at-risk communities, business communities, NGOs and civil society. These groups as stakeholders greatly determine the success of any disaster management intervention. In the public sector, success is measured by how successful a government is in delivering services to communities within its constituency (Lewis 2005:251; Lewis *et al*. 2001:7). Therefore, stakeholder relationships are very important, and it becomes imperative that all stakeholders including community, staff, funders and government accept and value the institution’s mission and support its strategies (Fill 2005:12–19; Lewis 2005:250). One way to achieve the above is to communicate in a two-way, participatory manner with all stakeholders involved. The importance of two-way or participatory communication to successfully managing key stakeholders in disaster risk management cannot be over emphasised. This type of communication style allows recipient communities to become part of the whole service delivery or disaster risk management process by allowing them to make decisions regarding the risk they face (identifying and analysing problems), as well as helping to develop possible risk reduction interventions. In order to achieve the goals in a disaster risk management context, it is therefore important that all role players communicate in a participatory manner, thus ensuring a good relationship between the numerous and complex stakeholders involved in the various processes and forums established to carry out the functions of disaster risk management.

As participation and communication are key aspects of stakeholder management in disaster contexts, the article will analyse the extent to which the MLM has worked in unison with and communicated with various stakeholders to carry out key functions of disaster management (as per the HFA and the *South African Disaster Management Act*) in relation to the sinkhole disaster risk within the municipality. Analysis will focus specifically on issues relating to the structures for stakeholder involvement in risk management, disaster risk assessment, training and awareness and early warning and response strategies. Before the results from the analysis can be elaborated upon, a brief outline of the study methodology is required.

## Methodology

A qualitative research design was selected for the study as it enabled the study of the phenomenon under investigation in its full complexity, portraying its multi-faceted forms whilst at the same time simplifying what was being studied and observed (Creswell 2003:250; Struwig & Stead 2007:190). The qualitative research method was suited to the context of the study as it required a deeper analysis of the opinion of three distinct groups of participants on the current implementation of the sinkhole risk reduction strategies within the MLM. These three groups were purposefully sampled for the information they could provide and can be outlined as follows.

### Ward councillors (in affected wards)

Councillors work with the community members on a daily basis, and are therefore likely to be aware of the interests and concerns of the community. The ward councillors represented all the wards (6) in the MLM which are affected by the occurrence of sinkholes.

### Community members (who live in areas that are affected by sinkholes)

In order to gain an individual as well as a shared experience and perception regarding the impacts of sinkholes and possible measures to mitigate the risk, total of 30 community members were targeted through focus group interviews.

### Senior disaster management official

The official is designated by the WRDM to implement disaster risk management services at the MLM. The official was selected to provide in-depth information regarding the development and implementation of sinkhole risk reduction and response strategies.

Semi-structured and focus group interviews were conducted with the various participants. Semi-structured interviews enabled the interviewer to tick responses on a pre-prepared schedule of questions whilst leaving sub-sections open for follow up questions, in case the interviewee wished to elaborate on a question and bring up additional lines of inquiry (Bell 2005:159; Berg 2007:20; Creswell 2003:251; Greener 2011:74; Guthrie 2010:108). According to Greener (2011) and Guthrie (2010), this method is most suitable for conducting an interview with individuals who can provide factual information regarding a specific topic. Consequently, this method of inquiry was applied with the official within the MLM disaster management department, in order to gain in-depth information on the mitigation as well as the response strategies to sinkhole formation. Additionally, semi-structured interviews were also conducted with the ward councillors of sections that were affected by the occurrence of sinkholes, in order to obtain the communities’ views regarding the impacts of sinkholes and what they think should be done in order to solve the problem. Ward councillors are well placed to give expert opinion on the current situation, as they are not just community members themselves but have been elected by their communities to represent their concerns at the local authorities. Ward councillors work with members of the community on a daily basis and are therefore likely to be aware of the interests and concerns of the community. It was crucial to interview councillors as traditionally they serve as links between the community and the local authority, and by interviewing them it would be possible to ascertain the current level of coordination between government officials, councillors and the communities which they serve.

Focus group interviews were also selected as a method of inquiry as they would enable the researcher to acquire individual and shared views regarding the impacts of sinkholes on the community, as well as their views on possible measures to reduce the risk (Langford & McDonough 2003:60–68; Morgan 1996:134). The six ward councillors were approached to help with the selection of focus group members, with five members being selected per ward to participate in the focus group. A total of 30 participants were selected on the basis of either having been effected by sinkhole formation, knowing someone that has been affected or has historical knowledge of sinkholes in the MLM.

The results from the questionnaires reveal many aspects about the current status of the dolomite risk reduction strategy in the MLM whilst also alluding to possible barriers such as deficiencies in stakeholder management that hamper the implementation of a strategy.

## Findings

Some of the key findings relating to stakeholder management will now be discussed under the themes of structures for stakeholder involvement in risk management, disaster risk assessment, training and awareness, and early warning and response.

### Structures for stakeholder involvement in risk management

Results of the interviews conducted with the wards councillors and community members revealed that the MLM does not have a committee that is specifically constituted to deal with sinkholes, even though the area is highly prone to the development of sinkholes.

Interviews conducted with the senior disaster official responsible for the implementation of disaster risk reduction at the MLM revealed that the WRDM is rendering disaster risk management services to the MLM, as the MLM does not have a disaster management centre of its own, because of human resource and financial constraints. Additionally, the WRDM does not have a committee which specifically addresses sinkhole-related matters. However, it has a Disaster Management Advisory Forum which addresses all hazards within the vicinity. The advisory forum comprises a wide array of stakeholders that can provide input on issues of risk management in the municipality. Some prominent stakeholders currently involved include municipal line departments, organised business, NGOs and Financing farmer producer organization (FPOs). Importantly, within the advisory forum, sinkholes are treated as a generic hazard that might occur in the municipality, but are not given a priority status above other hazards, even though the risk is quite extensive within the municipality.

### Disaster risk assessment

Community members and councillors revealed that the MLM does not conduct regular sinkhole risk assessments in the community. The community has also not been involved in the assessment processes that have been done in the past, as claimed by the MLM disaster management official (see paragraph below). This raises questions as to the validity and implementation of any existing sinkhole risk management strategy.

In contradiction to the response provided by the ward councillors and community members, the disaster official indicated that the district’s disaster management volunteers (from the district municipality) are deployed to conduct regular risk assessments under the supervision of the senior disaster official designated for the MLM. It can be argued that risk assessment, (as mentioned by the official), was either not conducted or conducted in such a way that both ward councillors and community members were not aware of any assessments being undertaken in their community. The lack of consultation with councillors and communities on the disaster risk could lead to ineffectiveness of how the WRDM manages sinkhole risk reduction in the MLM. For instance, programs to reduce the occurrence of sinkholes (the hazard component of the overall risk) might not be scaled to the correct level, as there might be a misunderstanding of the extensive nature of the sinkhole hazard. Additionally the strategy might not be focusing on the most affected communities, as the vulnerability assessment that underlines the risk assessment might be incomplete.

### Training and awareness

Councillors and communities were unanimous in stating that they have not received any training or awareness for the MLM regarding sinkhole risk reduction. As a result of a lack of risk reduction knowledge and capacity building, respondents felt that their risk of being affected by sinkholes is significantly exacerbated. It should be noted that the district has provided all the municipalities in its area with a dolomite risk strategy to effectively deal with sinkholes, but it has never provided training to affected communities regarding the implementation of the strategy. As a result, it can be argued that the success of the dolomite risk reduction strategy provided by the district to the municipality could be adversely affected by the lack of knowledge regarding its implementation.

The disaster official indicated that awareness campaigns were conducted in order to urge the community members to report the signs of sinkholes to the municipal offices or district disaster management centre. However, the response was contrary to the information provided by the ward councillors as well as the community members. Therefore, if ward councillors and community members were not aware of any awareness campaigns in their community, questions can be asked about the visibility of such campaigns, whether the training and awareness campaigns have been focused upon the most at-risk communities and the overall communication skills and stakeholder management capacity within the MLM.

### Early warning and response strategies

It also emerged that there is no sinkhole risk early warning system or sinkhole preparedness plan in place. It can be argued that because of the lack of early warning and preparedness plans, the community is more vulnerable to the impacts of sinkholes because the lack of knowledge and capacity to manage the risk effectively. Furthermore, in support to the response by ward councillors and community members, the disaster official was unable to answer whether the MLM has a sinkhole recovery strategy designed to reduce the impact of future sinkhole formation. Therefore, the absence of a sinkhole recovery strategy is likely to increase the susceptibility of the community to the impact of future sinkhole formations.

## Discussion

Various stakeholder management deficiencies are revealed by the research findings. In relation to *establishing and maintaining structures for stakeholder involvement* in risk management, it was found that the MLM does not have a disaster risk management centre or committee for sinkhole risk management in its area. Instead the duty of running a disaster management centre and disaster risk advisory committee resides with the West Rand District Municipality that is situated in Randfontein, approximately 50 km away. Although this situation (districts taking responsibility for disaster risk management functions at local municipality level) is allowed and even encouraged within the confines of the *South African Disaster Management Act*, it could cause a certain disconnect between district based disaster management officials and locally affected communities. This disconnect is often not helped by the fact that although a disaster management official can be appointed as a focal point for a specific area (the WRDM has appointed a focal point within its office to oversee the implementation of disaster risk management at the MLM), he or she could often also be appointed as a focal point for multiple areas, making it difficult to give detailed attention to specific at-risk areas. This disconnect seems to be present between communities, councillors and the disaster official, as they disagreed about the level of involvement of community stakeholders in major risk management functions and activities, such as disaster risk assessment and training and awareness campaigns.

The disconnect is further engrained by the current structure of the disaster risk management advisory forum and committee within the district, where there is no dedicated sinkhole risk management structure and key stakeholders are not brought together on a regular basis to discuss the extent of the sinkhole risk. As sinkholes pose a serious threat to inhabitants of the MLM, pro-active steps should be taken to establish a MLM based sinkhole risk committee that will include key stakeholders from government, communities and the business sector. At the very least, the district Disaster Management Advisory Forum should have a subcommittee set up to discuss sinkhole risks within the MLM and the whole district. However, such a subcommittee has not been set up, indicating a lack of a concerted effort to bring stakeholders together to specifically address the serious threat of sinkhole risk in the district. As a result of the location of the disaster management centre and lack of institutional support (committee) at the MLM or the WRDM, difficulties exist in bringing key stakeholders together, which in turn could affect the efficacy of risk reduction initiatives such as risk assessments, awareness campaigns (information sharing), early warning systems and implementation of disaster preparedness plans.

Some of the deficiencies caused by the lack of stakeholder management on the level of institutional arrangements already emerge upon review of the comments by community respondents (councillors or community) and the disaster management official for the area. Community respondents are unanimous in that they have never been part of *risk assessment* interventions within the MLM. The reason for the lack of involvement from affected communities in the risk assessment process lies with the fact that the WRDM disaster management volunteers were reported to have been used to conduct the assessments. This situation is problematic on several levels; however, the biggest concern lies with the fact that district level volunteers are gathered from all the local municipalities that form part of the district’s jurisdiction. This means that persons that are not necessarily familiar with the specifics of the sinkhole risk and the extent of the problem in the MLM are mandated to carry out an assessment with limited knowledge of the area under assessment. This could lead to misunderstanding of the scale of the risk that the MLM faces. Additionally, community stakeholders did not seem to be included in the assessment process by the district volunteers, as they were unaware of the assessment process in its totality. The lack of participation in processes and two-way communication to key community stakeholders means that the risk assessment process that forms the basis of possible sinkhole risk reduction strategies could be seriously flawed, as the total extent of the sinkhole risk that the MLM faces might not be fully understood by the WRDM. Additionally the use of district based volunteers to carry out the risk assessment, instead of those from affected communities, could place a strain on the trust relationship between the MLM and affected communities, as it could appear to the community that the municipality does not trust their experience of the risk and knowledge of the area enough to make them part of the assessment process. This lack of trust can prove devastating for community acceptance and buy-in for any sinkhole risk management strategy proposed by the municipality.

Subsequent to the risk assessment process that was mentioned above, a sinkhole risk strategy was developed for the MLM. However, from the perspective of community stakeholders, the content of this strategy has not been made part of *training and awareness campaigns*, leaving communities unaware of the extent of the risk and what mechanisms and processes have been put in place to reduce their susceptibility. The fact that community stakeholders that were interviewed indicated that they are not currently part of training and awareness campaigns does not mean that these have not been conducted on the part of the MLM. It does however point to shortcomings in identifying and including all at-risk communities in such interventions. The identification of critical stakeholders from at-risk communities will be a crucial first step towards improving the overall stakeholder management for the dolomite risk in the MLM. Once key stakeholders have been identified, the MLM can start to build a trust relationship with affected communities by regularly conducting house-to-house awareness campaigns or community-wide training initiatives on the sinkhole risk. The regular interaction with affected communities will illustrate the municipalities’ commitment to addressing the risk in partnership with affected communities, which in turn will strengthen the relationship between the two parties and contribute positively to the implementation of the MLM sinkhole management strategy.

Finally the data revealed that both the community stakeholders and disaster management officials are not aware of any response and early warning strategies currently in place to mitigate the possible impact of sinkhole formation in the community. Although the absence of such strategies should be of grave concern to the MLM and the WRDM, the lack of current mechanisms for response and early warning also provides an opportunity for greater cooperation and information sharing endeavours between disaster management officials and communities affected by dolomite.

## Conclusion

MLM has historically suffered financial and human losses because of sinkhole formation. Over time the extent of the problem has started to increase because of escalation in population growth, land tenure insecurity and pressure to provide basic services like water and sanitation. To address the increasing risk posed by sinkholes, the MLM has to urgently formulate an inclusive sinkhole risk reduction strategy. A key aspect to the success of such a strategy is the ability of the MLM to implement stakeholder management strategies. Improved stakeholder management would be characterised by improved two-way communication between the municipality and community stakeholders, fostering a relationship based upon trust and equality amongst stakeholders (in terms of inputs into planning for a comprehensive sinkhole risk management strategy), participation between a wide array of stakeholder groups affected by the sinkhole risk and a mutual commitment by all stakeholders to address the risk. The implementation of these stakeholder management characteristics between different organs of state (local, provincial, national government or line departments), at-risk communities, the business community, NGOs and civil society within the MLM will contribute to gaining greater insights into the extent of the risk and how best to manage the risk in an integrated and participatory fashion.

An evaluation of the current state of stakeholder management for sinkhole risk reduction in the MLM focusing on structures for stakeholder involvement in risk management, disaster risk assessment, training and awareness, and early warning and response revealed several deficiencies. In particular the study showed that in key disaster management activities and processes, such involvement in institutional bodies for risk management, disaster risk assessment, training and awareness is very limited or no stakeholder management is currently taking place. This lack of participation and inputs from sinkhole affected communities in the MLM means that the current district wide sinkhole risk reduction strategy could have fundamental flaws incorporated into its understanding of the extent of the problem and which communities are deemed priority areas for risk reduction interventions.

Additionally, no institutional avenue is currently available to affected communities to establish a regular two-way communication relationship with disaster management officials and other stakeholders about the sinkhole risk. This lack of communication leads to an added feeling of disconnect between affected communities and officials about the extent of the problem and who constitutes at-risk communities. The study also established that affected communities are also currently not involved in training and awareness programming relating to the sinkhole risk which they face.

What becomes clear from the issues mentioned is that there is almost a chronic lack of stakeholder management in the MLM when it comes to addressing sinkhole risk. The lack of stakeholder management has a negative impact upon the quality and comprehensiveness of the municipality’s sinkhole risk reduction strategy. This in turn limits the ability of the MLM to provide effective risk reduction strategies that will limit economic and human losses because of dolomite. Therefore, as a way forward, the MLM should make greater strides towards a comprehensive sinkhole risk reduction strategy that is inclusive of the views of multiple stakeholders, including affected communities. Additionally, institutional mechanism should be put in place to facilitate greater stakeholder management and involvement in key disaster risk management processes such as risk assessment, awareness and training, and early warning and response.
